# Complete Genome Sequence of Lentilactobacillus parabuchneri Strain KEM

**DOI:** 10.1128/MRA.01208-20

**Published:** 2021-05-20

**Authors:** Jongsun Park, Hong Xi, Yongsung Kim, Misun Kim

**Affiliations:** a InfoBoss, Inc., Gangnam-gu, Seoul, Republic of Korea; b InfoBoss Research Center, Gangnam-gu, Seoul, Republic of Korea; c MisunBio, Seocho-gu, Seoul, Republic of Korea; University of Maryland School of Medicine

## Abstract

Korean effective microorganisms (KEM) comprise a commercial microbial complex originating from the microorganisms found in traditional Korean fermented foods, including kimchi. Using next-generation sequencing (NGS) technology, the complete genome sequence of Lentilactobacillus parabuchneri, considered a major species of KEM, was assembled. *L. parabuchneri* strains display large numbers of single nucleotide polymorphisms (SNPs) and indels, including gene deletions.

## ANNOUNCEMENT

Korean effective microorganisms (KEM) comprise a commercial microbial complex originating from the microorganisms found in traditional Korean fermented foods, including kimchi. This complex can remove toxic materials from trash and promote plant growth when used in soil. The major bacterial species in KEM include *Lactobacillus* species based on microbial community analysis of 16S rRNA; however, no additional scientific evidence is available. KEM is expected to contain multiple bacterial species, such that genome sequencing technologies can be applied to obtain complete genomes of the bacterial species in KEM. Recently, *Lactobacillus* was relocated to the genus *Lentilactobacillus* based on the polyphasic approach (1). Here, we report the complete genome sequence of Lentilactobacillus parabuchneri, considered a major species of KEM, which also plays an important role in cheese ripening by eye formation in cheese (2).

Total DNA was extracted from 1 ml KEM liquid sample (InfoBoss Cyber Herbarium, IN; M. Kim; IBS-00007) using a DNeasy plant minikit (Qiagen, Hilden, Germany). Genome sequencing was conducted using the HiSeq 2000 platform at Macrogen, Inc., (South Korea), obtaining 50.55 million 101-bp paired-end reads via a 350-bp insert paired-end library constructed with a TruSeq Nano DNA library preparation kit (Illumina, San Diego, CA) following the manufacturer’s recommendations. *De novo* assembly was conducted using Velvet v1.2.10 (3) with the minimum depth option set to 20×, after filtering the raw reads using Trimmomatic v0.33 (4) in the Genome Information System (GeIS) environment (1, 5, 6). Contig sequences from the Velvet assembly were selected based on BLASTN results from the nonredundant (NR) database with homology to *L. parabuchneri* (nucleotide identity above 90%). GapCloser v1.12 (7), BWA v0.7.17 (8), SAMtools v1.9 (9), and Geneious R11 v11.1.5 (Biomatters Ltd., Auckland, New Zealand) were used for filling the gaps, identifying that it was a circular genome, and validating the assembly. All bases were confirmed using BWA v0.7.17 (8) and SAMtools v1.9 (9). Default parameters were used for all software unless otherwise specified. CheckM was used to check the quality of the assembled genome sequence (10) with a completeness of 98.71%, contamination of 0.81%, and strain heterogeneity of 0.00%. The coverage of the assembled genome is 40.78×. Genome annotation was conducted using the NCBI Prokaryotic Genome Annotation Pipeline (11).

The complete genome sequence of *L. parabuchneri* strain KEM is 2,530,879 bp long, with a GC content of 43.6%; it is shorter than the previously sequenced *L. parabuchneri* genome (2,600,578 bp; GenBank accession number CP018796) by 69,699 bp. Based on the pairwise alignment of both *L. parabuchneri* genome sequences, 8,402 single nucleotide polymorphisms (SNPs) and 427 indels (88,287 bp) were identified, accounting for 0.332% and 3.489%, respectively. In total, 2,228 protein-coding genes, 15 rRNAs, 60 tRNAs, and 2 noncoding RNAs (ncRNAs) were predicted, which is 196 fewer protein coding genes than in the genome submitted under GenBank accession number CP018796, a difference caused by several large deletions. For example, the transposase DDE domain protein coding DNA sequence (CDS) (APR07110.1) in CP018796 was missing from our genome.

We expect that our complete genome sequence will provide clues for understanding the functionality of this species in KEM. In addition, our genome sequence displays intraspecific variations, including several gene-level deletions, as part of the genomic diversity of *L. parabuchneri*.

### Data availability.

The Lactobacillus parabuchneri strain KEM whole-genome sequencing project has been deposited at DDBJ/ENA/GenBank under the accession number CP062473, BioProject accession number PRJNA665775, and BioSample accession number SAMN16268313. The raw sequences were deposited under the accession number SRR12717129.
